# Percutaneous transhepatic arterial access for coil embolization of hepatic artery infusion pump-associated bleeding

**DOI:** 10.1186/s42155-024-00515-w

**Published:** 2025-01-09

**Authors:** Anuj K. Dutta, Vishal Shankar, Ernesto G. Santos, Brett Marinelli, Erica S. Alexander, Vlasios S. Sotirchos, Ken Zhao

**Affiliations:** 1 Albert Einstein College of Medicine, New York, NY 10461, USA; 2 Department of Radiology, Memorial Sloan Kettering Cancer Center, New York, NY 10065 USA

**Keywords:** Hepatic artery infusion pump, Transhepatic arterial access, Hepatic arterial bleeding

## Abstract

**Background:**

Hepatic artery infusion pump (HAIP) chemotherapy is a locoregional treatment for intrahepatic malignancies. HAIPs are surgically implanted, and the catheter tip is typically inserted into a ligated gastroduodenal artery stump. Potential complications at the catheter insertion site include dehiscence, pseudoaneurysm or extravasation, and adjacent hepatic arterial stenosis and thrombosis. Bleeding complications can be treated with stent-graft placement or coil embolization across the injury site, typically with standard antegrade arterial approach into the hepatic arterial system by transfemoral or transradial access. However, in cases where an antegrade approach is not possible, alternative methods are necessary.

**Case presentation:**

A patient presented with an enlarging hematoma due to bleeding at the gastroduodenal artery HAIP catheter insertion site. Emergent angiography revealed concomitant common hepatic artery occlusion and retrograde perfusion of the GDA via tortuous, diminutive hepatic collaterals which precluded standard antegrade approach. Collateral inflow from the dorsal pancreatic artery was utilized to opacify the right hepatic artery. The segment 5 hepatic artery was percutaneously accessed under fluoroscopic guidance, and microcoils were deployed both proximal and distal to origin of the gastroduodenal artery. The patient remained stable throughout the postoperative period and was discharged after an otherwise uneventful admission. Follow-up computed tomography demonstrated resolution of the hematoma and no bleeding or biliary complication from transhepatic access.

**Conclusions:**

This report highlights the safety and efficacy of percutaneous transhepatic arterial access for endovascular management of HAIP associated bleeding at the gastroduodenal artery when standard antegrade interventions cannot be performed. Interventional radiologists caring for patients with HAIPs should be familiar with their potential complications and the range of techniques required for management.

## Background

Hepatic artery infusion pump (HAIP) chemotherapy is a locoregional treatment for intrahepatic malignancies, both primary and metastatic. HAIPs utilize hepatic first-pass metabolism and permit high-dose administration of select chemotherapeutics [1]. HAIPs are surgically implanted, and the catheter is typically inserted within a ligated gastroduodenal artery (GDA) [1]. During implantation, the hepatic artery and the GDA are skeletonized by ligation of extrahepatic branches, such as the right gastric artery and suprapyloric branches, to prevent extrahepatic mis-perfusion during administration of HAIP chemotherapy and resultant injury to the bowel or pancreas [1]. Potential complications include pseudoaneurysm or extravasation at the catheter tip insertion site, and adjacent arterial stenosis and thrombosis of the common and proper hepatic arteries. HAIP catheter associated bleeding is usually treated with stent-graft placement or coil embolization across the site of arterial injury [1]. We report a case of concomitant HAIP associated bleeding and common hepatic artery (CHA) occlusion. The CHA occlusion and the patient’s post-surgical vascular anatomy, which limited the options for collateral navigation, precluded endovascular treatment via a standard antegrade approach with transfemoral or transradial access, necessitating percutaneous transhepatic arterial access. This retrospective report was institutional review board approved.

## Case presentation

**Fig. 1:**
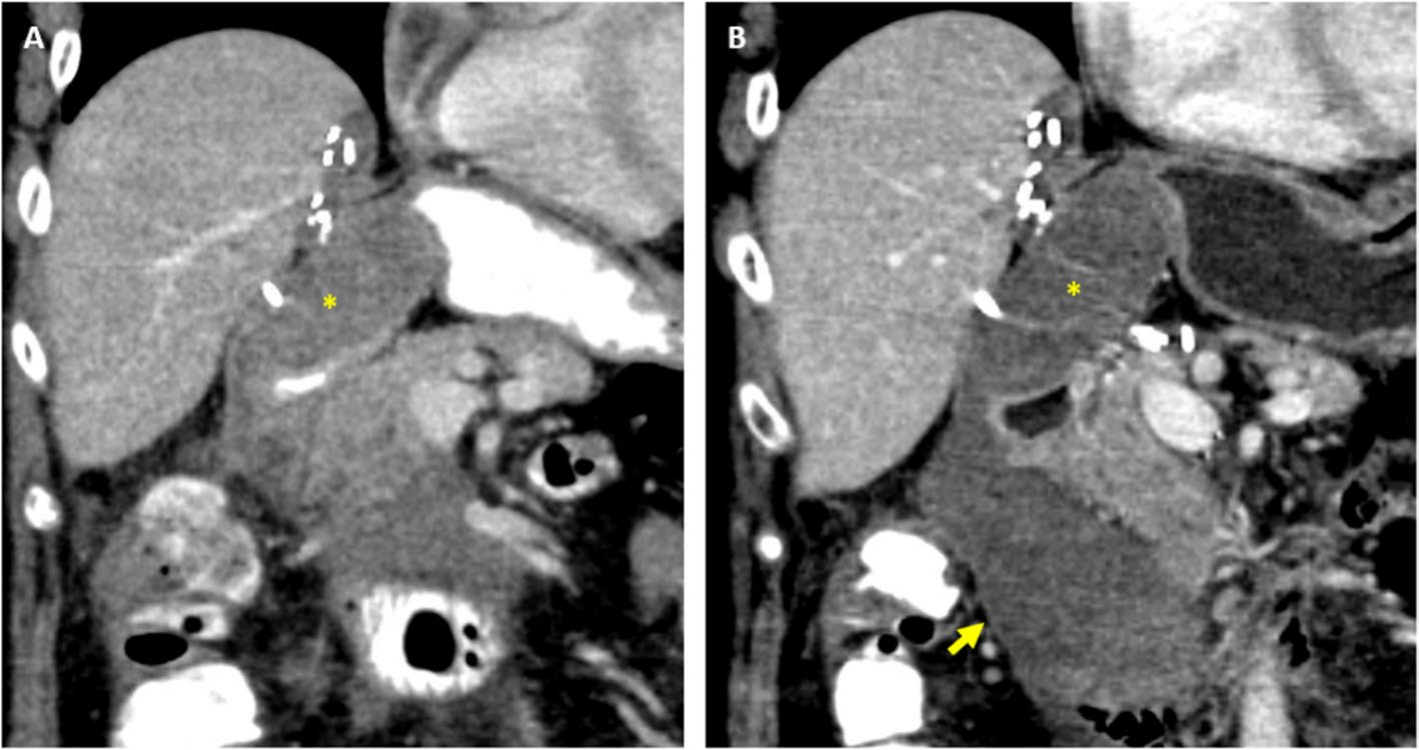
Pre-procedural contrast-enhanced CTs. **A** Initial coronal image demonstrates a hematoma (asterisk) adjacent to the HAIP catheter insertion at the GDA. **B** Short interval follow-up coronal image demonstrates enlargement of the hematoma, most prominently at the inferior aspect (arrow)

A patient with rectosigmoid adenocarcinoma and hepatic metastases presented with right lower quadrant pain. About 4 years prior, the patient had underwent low anterior resection, left hepatic lobectomy, and HAIP implantation for HAIP chemotherapy. Initial contrast-enhanced CT scan showed a hematoma adjacent to the HAIP catheter insertion (Fig. 1A). No active extravasation was seen. However, the patient’s symptoms worsened, hemoglobin declined from 11.8 to 8.7 g/dL, and a short interval follow-up contrast-enhanced CT scan demonstrated enlargement of the hematoma consistent with bleeding at the GDA remnant (Fig. 1B).

**Fig. 2:**
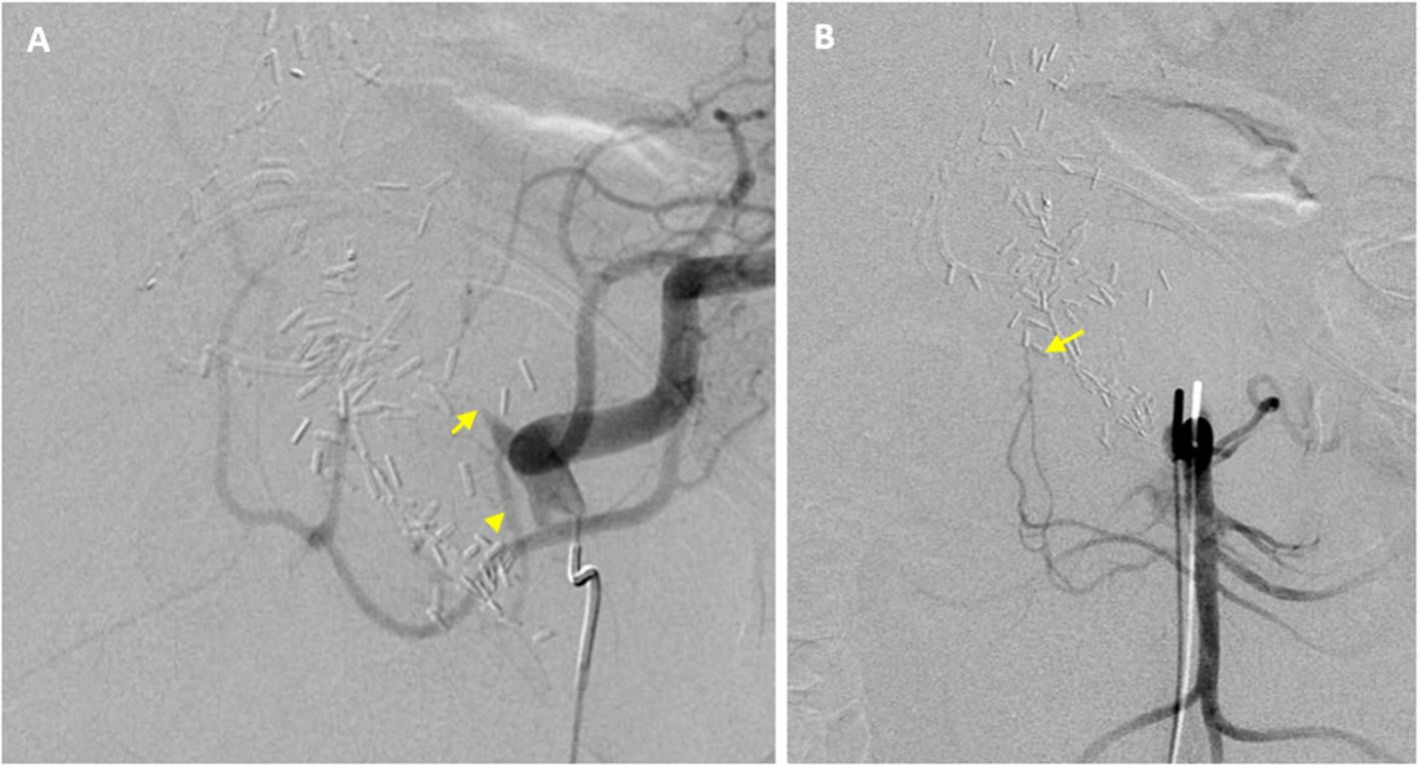
Initial angiograms. **A** Celiac angiogram demonstrating an occluded CHA (arrow). The DPA (arrowhead) is seen arising from the proximal aspect of the CHA. **B** Superior mesenteric artery angiogram demonstrating truncation of the inferior pancreaticoduodenal artery (arrow)

**Fig. 3:**
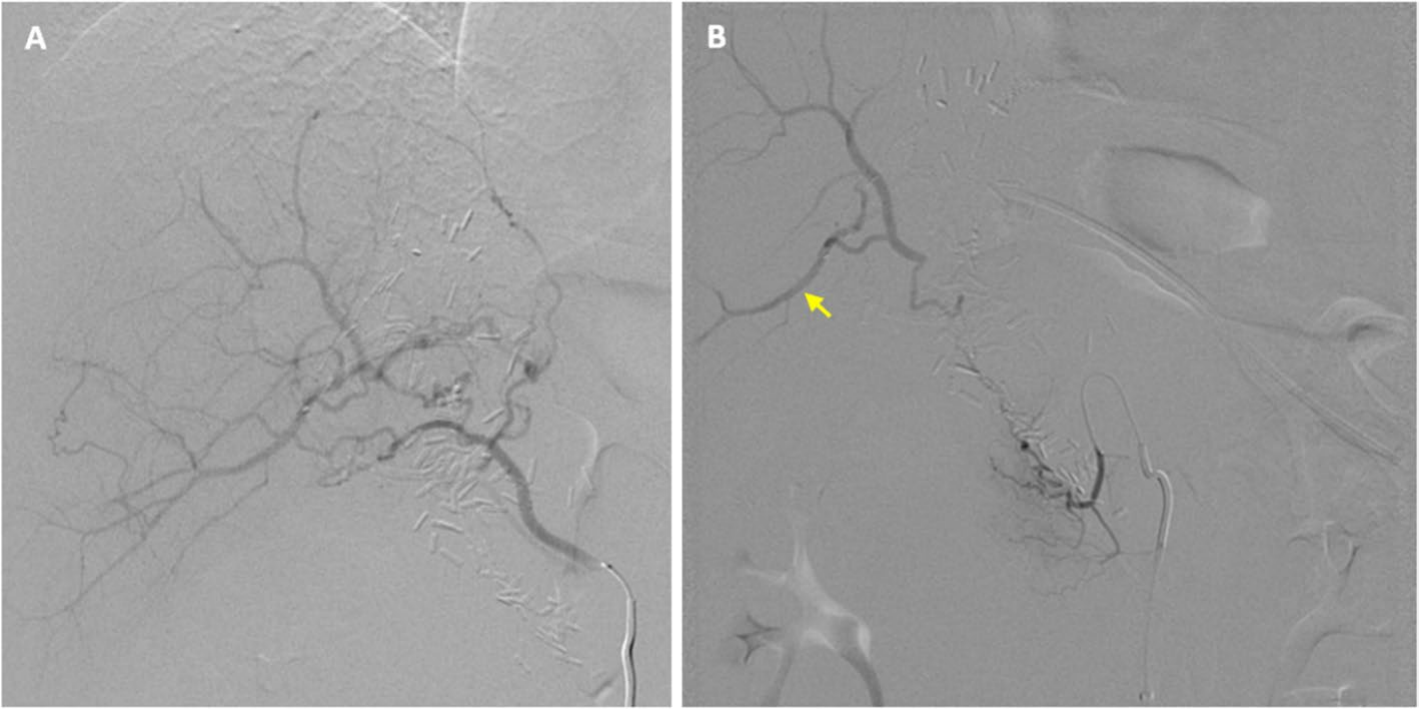
Hepatic collaterals. **A** Right inferior phrenic artery angiogram and (**B**) DPA angiogram demonstrating collateral perfusion of the hepatic arteries. The segment 5 hepatic artery (arrow) was targeted for transhepatic arterial access

**Fig. 4:**
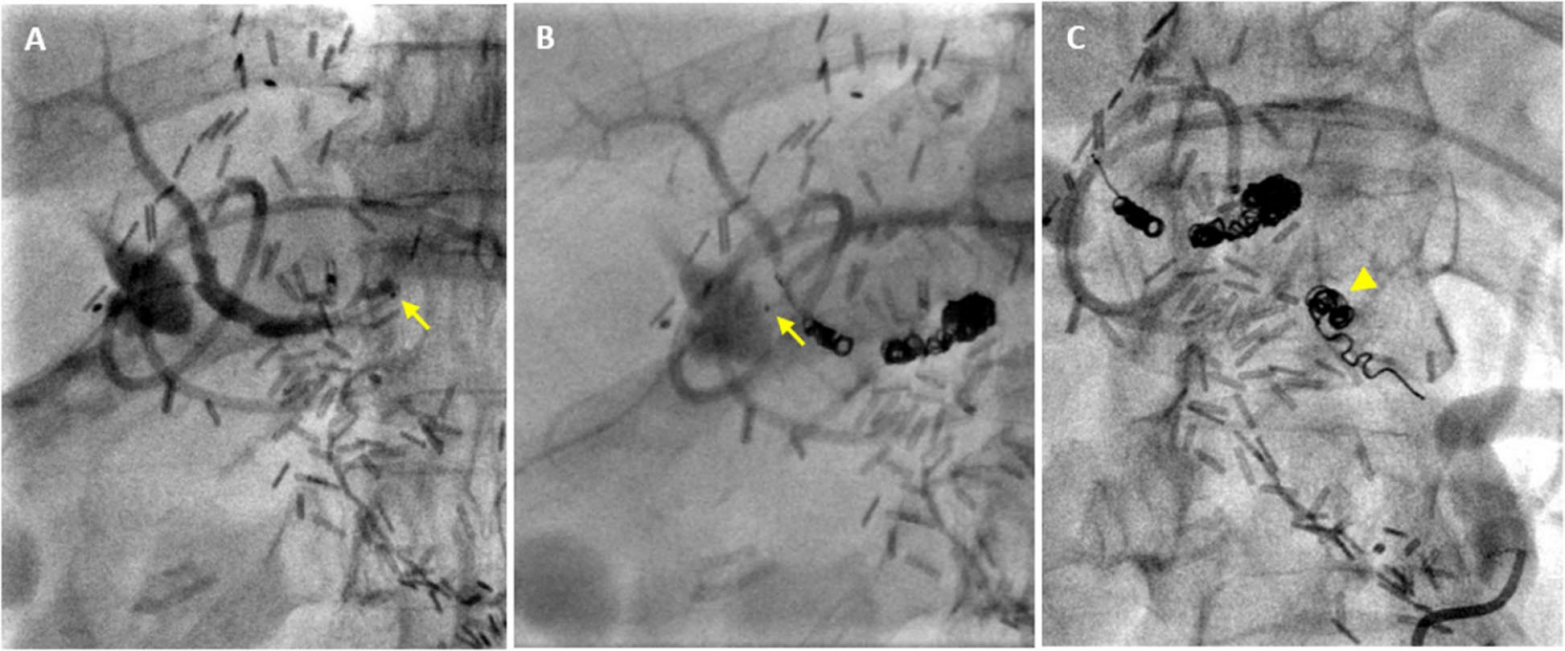
Fluoroscopic images of coil embolization. **A** PHA angiogram obtained after advancing the microcatheter (arrow) as far retrograde as possible. Note hepatic contrast staining from attempts at percutaneous arterial access. **B** Segment 5 angiogram obtained after PHA coiling and retraction of the microcatheter (arrow), demonstrating stasis at the coil pack.** C** Celiac angiogram demonstrating stasis at the CHA stump coil pack (arrowhead)

The patient emergently went to the interventional radiology suite for angiography and embolization. Celiac angiogram demonstrated an occluded CHA which could not be crossed to access the GDA remnant (Fig. 2A). Superior mesenteric artery angiogram showed truncation of the inferior pancreaticoduodenal artery without visualization of the pancreaticoduodenal arcade, findings consistent with surgical ligation of the distal GDA (Fig. 2B). Thorough investigation to find the source of “back-door” inflow to the GDA revealed hepatic arterial collateralization with the right inferior phrenic and dorsal pancreatic arteries (DPA) (Fig. 3), however, the collaterals were too small and tortuous to navigate with a microcatheter. Intraprocedural ultrasound visualized continued enlargement of the hematoma. After discussion with hepatobiliary surgery and the patient’s healthcare proxy regarding risks and benefits, the decision was made to proceed with percutaneous transhepatic arterial access (Fig. 4).

Initial attempts to establish transhepatic access into a branch of the right hepatic artery using a 21 G needle (Percutaneous entry thinwall needle; Cook Medical, Bloomington, Indiana) and ultrasound guidance were unsuccessful due to poor arterial visualization. Subsequent attempts were performed using fluoroscopic guidance, aiming for the segment 5 hepatic artery while it was opacified by collateral inflow during contrast injection of the DPA (Fig. 3B). The segment 5 artery coursed linearly, facilitating in-line needle orientation with the vessel and maximizing the likelihood of successful puncture. After contrast injection through the needle confirmed intraluminal access, a 0.016’’ hydrophilic microwire (Meister; Asahi Intecc, Irvine, California) was advanced into the right hepatic artery. A 2.0 Fr microcatheter (Truselect; Boston Scientific, Marlborough, Massachusetts) was advanced over the wire and positioned as far retrograde within the proper hepatic artery (PHA) as possible (Fig. 4A). Embolization of the PHA was performed with 3 and 4 mm diameter fibered detachable microcoils (Embold; Boston Scientific, Marlborough, Massachusetts), closing the “back-door” inflow to the GDA (Fig. 4B). The transhepatic arterial access tract was embolized with N-butyl cyanoacrylate glue (Histoacryl; Braun, Bethlehem, Pennsylvania) mixed with lipiodol in a 1:1 ratio that was slowly injected during microcatheter withdrawal. Establishment of transhepatic arterial access required approximately 45 min and numerous attempts.

The proximal aspect of the CHA stenosis was accessed via standard antegrade approach through the right common femoral artery. A 2.4 Fr microcatheter (Progreat; Terumo, Somerset, New Jersey) was advanced into the truncated CHA. Embolization was performed with 4-mm diameter fibered detachable microcoils (Embold; Boston Scientific, Marlborough, Massachusetts) deployed within the truncated CHA. This was done to definitively close the “front-door” and prevent potential recurrence of bleeding should the CHA recanalize (Fig. 4C).

The patient remained stable during the postoperative period and was discharged after an otherwise uneventful admission. Follow-up CT scans demonstrated progressive decrease in the size of the hematoma, with eventual complete resolution, and no evidence of bleeding or biliary complication from the transhepatic access.

## Discussion

In the present case, occlusion of the CHA and a lack of navigable hepatic collaterals prevented standard antegrade approach with transfemoral or transradial access. The decision to proceed with transhepatic arterial access was made given ongoing bleeding and after intraprocedural discussion with hepatobiliary surgery.

This is a technically challenging method of arterial access which was chosen as the final attempt at endovascular management. Potential complications from hepatic punctures during attempts at access include hemorrhage, arterio-biliary/biliary-venous fistula, and biliary injury which may result in bile leak or biloma. Percutaneous transhepatic arterial access has been described in few prior reports [2–4]. Huf et al. and Paz-Fumagalli et al. utilized ultrasound guidance and report the hepatic arterial puncture as the most challenging technical hurdle during their procedures. A fluoroscopically guided puncture was described by Tamura et al., who utilized angiography and an existing percutaneous biliary drainage catheter for guidance [4].

Our initial attempts at transhepatic arterial access under ultrasound guidance were unsuccessful due to inability to visualize the target. Subsequently, angiography opacified the target vessel and permitted successful fluoroscopic puncture. The 45 min required to puncture and catheterize the segment 5 hepatic artery is similar to what has previously been reported [3].

## Conclusion

Interventional radiologists involved in the care of HAIP patients should be aware of the associated complications and techniques which may be necessary in select situations. While a rare occurrence, given the potential for complications at the HAIP insertion site and operative skeletonization of the GDA and hepatic arteries, HAIP patients are more likely to require alternative methods for endovascular management of bleeding complications. Percutaneous transhepatic arterial access for embolization of bleeding may represent a feasible and safe alternative approach when conventional antegrade methods are not possible.

## Data Availability

Not applicable.

## Abbreviations

HAIP: Hepatic Artery Infusion Pump
GDA: Gastroduodenal Artery
CHA: Common Hepatic Artery
DPA: Dorsal Pancreatic Artery
PHA: Proper Hepatic Artery
